# Secondary azoospermia after a successful natural pregnancy: a primary prospective study

**DOI:** 10.1186/s12610-024-00227-0

**Published:** 2024-08-06

**Authors:** Amr Elahwany, Hisham Alahwany, Hesham Torad, David Ramzy, Elshaimaa Ahmed Fahmy Aboelkomsan, Sameh Fayek GamalEl Din

**Affiliations:** 1 Department of Andrology and STDs Kasr Al-Ainy, Sexual medicine and STIs department, Faculty of Medicine, Cairo University, Al-Saray Street, El Manial, Cairo, 11956, Egypt; 2 Nile center for IVF, Cairo, Egypt; 3 Department of Urology, Faculty of medicine, Cairo University, Cairo, Egypt; 4 Pathology department, School of medicine, Newgiza University, Giza, Egypt

**Keywords:** Secondary azoospermia, Natural pregnancy, microTESE, Azoospermie secondaire, Grossesse naturelle, microTESE

## Abstract

**Background:**

To date, there is a lack of studies conducted on males with secondary azoospermia as a potential cause of male infertility who had previously fathered children through natural conception. The current study aims to investigate the potential causes of secondary azoospermia as a presentation of male infertility as well as the prognostic factors that can impact sperm retrieval rate (SRR) while undergoing microdissection testicular sperm extraction (microTESE).

**Results:**

Thirty two patients were recruited from the andrology outpatient clinic from August 2023 till January 2024. The mean age of the patients was sixty-two years old. All patients had varicoceles. Twenty seven patients (84%) had palpable varicocele grade 2 and 3 on both sides. Further multivariate logistic regression analysis of the significant factors in the univariate regression revealed that younger age (OR 0.7, 95% C.I. 0.7-1.0, *p* = 0.03) and having a history of coronary artery disease (CAD) were predictable factors for negative TESE outcome (OR 123.1, 95% C.I. 3.2-4748.5, *P* = 0.01).

**Conclusion:**

It appears that the etiopathogenesis of secondary azoospermia are multifactorial. Varicocele and CAD are major factors to be considered. Future studies should be implemented deploying larger pools of patients suffering from the same condition to affirm the findings of this primary study.

## Résumé

**Contexte:**

À ce jour, il existe un manque d’études menées chez des hommes atteints d’azoospermie secondaire comme cause potentielle d’infertilité masculine, alors qu’ils avaient déjà engendré des enfants par conception naturelle. La présente étude vise à étudier les causes potentielles de l’azoospermie secondaire en tant que présentation de l’infertilité masculine, ainsi que les facteurs pronostiques qui peuvent avoir un impact sur le taux de récupération des spermatozoïdes (SRR) lors de l’extraction de spermatozoïdes testiculaires par microdissection (microTESE).

**Résultats:**

Trente-deux patients ont été recrutés dans la clinique ambulatoire d’Andrologie d’août 2023 à janvier 2024. L’âge moyen des patients était de soixante-deux ans. Une varicocèle était présente chez tous les patients. Vingt-sept patients (84%) présentaient une varicocèle palpable de grade 2 et trois une varicocèle bilatérale. Une analyse par régression logistique multivariée des facteurs significatifs lors de la régression univariée a révélé qu’avoir un âge plus jeune (OR 0,7, IC à 95 % 0,7-1,0, *p* = 0,03) et des antécédents de maladie coronarienne (coronaropathie) étaient des facteurs prédictifs d’un résultat négatif à la microTESE (RC 123,1,95 % IC 3,2-4748,5, *p* = 0,01).

**Conclusions:**

Il apparaît que l’étiopathogénie de l’azoospermie secondaire est multifactorielle. La varicocèle et la coronaropathie sont des facteurs majeurs à prendre en compte. De futures études devraient être mises en œuvre en déployant des groupes de patients plus nombreux présentant la même affection pour confirmer les résultats de cette étude primaire.

## Introduction

Secondary infertility is the inability to conceive after one year of regular, unprotected intercourse following term childbirth without using assisted reproductive techniques (ART) or fertility medications [1]. Around one-third of cases are due to male factors [2]. Male factor infertility can result from several etiologies, including varicocele, which is the cause of nearly 81% of cases of secondary male infertility [3]. Aging also has a progressive impact on spermatogenesis through the cumulative deleterious effect of reactive oxygen species on spermatogenic cells together with amyloidosis in the testis [3, 4]. Environmental factors such as food pesticides and lifestyle factors such as smoking and eating habits affect spermatogenic cells and increase DNA fragmentation [5, 6]. Also, caffeinated beverages high consumption causes spermatogenic arrest and affects Sertoli cell function [5, 6]. There are several genetic causes of azoospermia as a primary cause of infertility, including Y chromosome microdeletions, Klinefelter, Noonan, and Kallmann syndromes, among others [7]. Secondary infertility projected to 3.3 million in 2006 accounting for six out of ten infertility cases [8]. Nevertheless, the topic of secondary azoospermia was rarely discussed. Thus, there is a lack of studies conducted on infertile males with secondary azoospermia who had previously fathered children through natural conception. The current study aimed to investigate the potential causes of secondary azoospermia as a presentation of male infertility as well as the prognostic factors that could impact sperm retrieval rate (SRR) while undergoing microdissection testicular sperm extraction (microTESE).

## Patients and methods

Thirty two patients were recruited from the andrology outpatient clinic from August 2023 till January 2024. The institutional review board approved the work (N-265-2023) that conforms to Helsinki declaration 2013 [9]. All the participants were aware about the purpose of the study and signed an informed consent prior to joining the study.

### Inclusion criteria of the patients

Males complaining of secondary azoospermia after fathering children through natural conception were included in the study.

### Exclusion criteria of the patients

Patients with a history of testosterone or steroid abuse, bilateral undescended testes, bilateral epididymorchitis, untreated gonorrhea, chemotherapy or obstructive azoospermia were excluded. Finally, cases of cryptozoospermia and Y chromosome microdeletion were excluded.

All patients were subjected to the following:

Medical and operative histories were obtained. Patients underwent a thorough general and local examinations. Semen analysis was done according to the guidelines of the 5th edition of WHO (2010) [10]. Two semen analyses were obtained on two different occasions with an abstinence period of 2 to 7 days [11]. All semen analyses of the patients showed normal volume as well as normal physical characteristics. However, all of them showed absence of sperms.

Five cc early morning blood sample (before 11 AM) was withdrawn for full hormonal profile including blood follicle stimulating hormone (FSH), leutinizing hormone (LH), total testosterone, estradiol, and prolactin that were evaluated using chemiluminescence immunoassay (CLIA) technique (1.5–14 mIU/ml for FSH, 1.5–8 mIU/ml for LH, 2.5–17 ng/ml for prolactin, 2.4–8.3 ng/ml for total testosterone, and 20–47 pg/ml for estradiol). All assays were executed utilizing Cobas E411 immunoassay analyzer (Roche Diagnostics GmbH, Mannheim, Germany). If varicocele was detected during genital examination, a scrotal duplex study was done to determine the degree of varicocele. The diagnosis of non obstructive azoospermia (NOA) was initially made if FSH level was elevated together with repeated azoospermia and empty epididymis on examination. MicroTESE was executed through a median raphe incision followed by delivery of one testis. A transverse incision was made in the tunica albugenia by avoiding subtunical vessels injury. The testicular tissue was observed using high magnification 25x surgical microscope (M320, Leica, Germany). All surgical procedures were done by an expert andrologist. Dilated tubules were identified and biopsied. Testicular tissue was examined by two senior embryologists throughout the study. If the initial search revealed the presence of sperms, the operation was terminated and the testis was closed. If the sperm search was negative, the contralateral testis was opened. Testicular tissue was obtained for histopathology that was preserved in Bouin’s solution. Histopathological examination was done by an expert pathologist. Based on the morphological pattern, histopathology was classified into: hypospermatogenesis, maturation arrest, Sertoli cell only (SCO), tubular hyalinization and mixed pattern [12].

Finally, patients were divided into two groups based on the results of the microTESE and the two groups were compared based on their clinical, demographic, hormonal, and testicular histopathological findings.

### Statistical analysis

The SPSS 22nd version was used to do the statistics. For the initial features of the patients, descriptive statistics were done. Univariate and multivariate logistic regression analyses and chi square tests were used to find predictors for microTESE data and histopathological diagnosis.

## Results

A total of 32 patients were enrolled in our study. The mean age of the patients was sixty-two years old (Table 1). The duration of infertility ranged from one to twenty years after delivery of their youngest child (Table 1). Average sibling’s age was twenty-one years old. 46% of the cases smoked tobacco, 46% of the cases had diabetes mellitus, half of them had hypertension and one third had a history of coronary artery disease (CAD) (Table 1). All patients had varicoceles where 27 patients (84%) had palpable varicocele grade 2 and 3 on both sides (Table 1). The mean FSH and LH were higher than the upper normal range (15.7 mIU/mL and 10.2 IU/L, respectively) (Table 1). Total testosterone was less than 3 ng/ml in more than half of the patients (seventeen patients), with a mean below the lower normal range limit (2.5 ng/ml) (Table 1). Patients who had positive microTESE results were significantly older (mean 65.6 ± 7.1, *P* = 0.030), less likely to smoke (71.40% of non-smokers *P* = 0.004), less likely to have a history of CAD. Additionally, 85.7% of the cases had hypospermatogenesis (*P* < 0.001) (Table 2).

**Table 1:** Shows demographic and clinical and hormonal and testicular histology characteristics of the included patients

	Mean ± SD	Range
Patient's age (years)	62.1± 10.5	38-83
Duration of infertility (years)	4.7 ± 4.4	1-20
Age of youngest child (years)	21.8± 11	4-42
Diabetes		
No	17	53.1%
yes	15	46.9%
Smoking		
Non-smoker	17	53.1%
Smoker	15	46.9%
FSH ^a^ ng/ml	15.7± 7.8	6-44
LH ^b^ ng/ml	10.2± 4.3	6-23
Total testosterone ng/ml	2.5± 0.9	1-5
Varicocele		
No	0	0.0%
Grade 1	5	15.6%
Grade 2	17	53.1%
Grade 3	10	31.3%
microTESE^c^ outcome		
No sperms	11	34.4%
Sperm retrieved	21	65.6%
Testicular histopathology		
C1	6	18.8%
Hypospermatogenesis	18	56.3%
SCO	4	12.5%
SCO+Focus	4	12.5%
Hypertension		
No	16	50.0%
Yes	16	50.0%
Coronary artery disease		
No	23	71.9%
Yes	9	28.1%
Pregnancy		
No pregnancy	22	68.8%
pregnancy	10	31.3%

N.B. ^a^follicle stimulating hormone; ^b^leutinizing hormone; ^c^*microTESE* microsurgical testicular sperm extraction

50% of patients had spermatogenic arrest or Sertoli cell only (SCO) in histopathology (Table 2). All patients who had hypospermatogenesis and SCO with spermatogenic foci were older than 50 years old (Table 2). Moreover, chi square test was performed to see if there was an association between patient’s age and histopathological diagnosis and found that males older than 50 years old are more likely to give pathology results of hypospermatogenesis or SCO with spermatogenic foci (*p* = 0.001). Additionally, univariate logistic regression analysis was performed to see which of the patients’ factors influenced the microTESE outcome. The age of the patients was inversely associated with microTESE outcome (OR 0.9, 95% C.I. 0.8-1.0, *p* = 0.02). Smoking (OR 11.2, 95% C.I. 1.85–68.1, *p* = 0.008) and a history of CAD (OR 7.2, 95% C.I. 1.3–39.6, *P* = 0.02) were associated with an increased risk of a negative microTESE outcome. Further multivariate logistic regression analysis of the significant factors in the univariate regression revealed that younger age (OR 0.7, 95% C.I. 0.7-1.0, *p* = 0.03) and having a history of CAD were predictable factors for negative micro-TESE outcome (OR 123.1, 95% C.I. 3.2-4748.5, *P* = 0.01). However, smoking had no significant association with the micro-TESE outcome (OR 1.8, 95% CI 0.1–38.4, *p* = 0.6).

**Table 2:** Shows the relationship between sociodemographic characteristics and laboratory findings and microTESE outcome among the participants

	microTESE^d^ outcome				*P* value
	negative		positive		
	Mean ± SD	Range	Mean ± SD	Range	
Patient’s age (Years)	55.4± 12.9	38-76	65.6± 7.1	52-83	0.030^e^
Duration infertility (Years)	7.7± 6.4	1-20	3.2± 1.5	1-6	0.113
Age of youngest child (Years)	18.5± 13.6	4-42	23.4± 9.3	9-42	0.238
FSH ng/mL	16.8± 8.8	6-33	15.1± 7.3	10-44	0.667
LH ng/mL	11.5 ± 4.9	6-23	9.6 ± 4	6-23	0.271
Total testosterone ng/ml	2.6± 1.3	1-5	2.6± 0.8	1.1-4	0.815
	N	%	N	%	
Diabetes					
No	5	45.50%	12	57.10%	0.529
Yes	6	54.50%	9	42.90%	
Smoking					
Non-smoker	2	18.20%	15	71.40%	0.004^e^
Smoker	9	81.80%	6	28.60%	
varicocele					
No	0	0.00%	0	0.00%	0.422
Grade 1	1	9.10%	4	19.00%	
Grade 2	5	45.50%	12	57.10%	
Grade 3	5	45.50%	5	23.80%	
Testicular histopathology					
C1^a^	6	54.50%	0	0.00%	<0.001^e^
Hypospermatogenesis	0	0.00%	18	85.70%	
SCO^b^	4	36.40%	0	0.00%	
SCO^b^ + spermatogenic focus	1	9.10%	3	14.30%	
Hypertension					
No	6	54.50%	10	47.60%	0.710
Yes	5	45.50%	11	52.40%	
CAD^c^					
No	5	45.50%	18	85.70%	0.016^e^
yes	6	54.50%	3	14.30%	

N.B ^a^primary spermatocyte arrest; ^b^Sertoli cell only syndrome; ^c^*CAD* coronary artery disease; ^d^*microTESE* microsurgical testicular sperm extraction; ^e^*p* value was calculated using chi square test

## Discussion

The current study demonstrated that the included patients who previously fathered children by natural conception were currently azoospermic. Unfortunately, all included cases did not have any semen report prior to fathering their children. Furthermore, patients with positive microTESE outcome were compared to those with negative microTESE outcome regarding age, varicocele grade, diabetes milletus, CAD, smoking and hypertension. Our study had revealed that ageing and non smoking were associated with favourable microTESE outcome.

In a similar trend, Li et al. (2018) had shown that age might have a predictive value for successful sperm recovery [13]. Quite the reverse, several studies had shown that ageing as well as life style might have detrimental impact on spermatogenesis attributed to progressive damage by oxidative stress [4, 6, 14, 15]. Remarkably, there is no consensus on the cut off value for advanced paternal age [14, 16–19]. In the same context, Ramasamy et al. (2014) and Amer et al. (2019) did not find any correlation between ageing and SRR [20, 21]. However, it should be mentioned that a critical review stressed on the fact that male age being a key determinant for the health of the offspring rather than the inability to conceive [22]. Moreover, the current study had demonstrated that CAD was associated with unfavourable microTESE outcome. This finding could be explained as follow. Firstly, Haverich (2017) postulated that atherosclerosis was a microvascular disease rather than a large-vessel disease [23]. Thus, testicular circulation might be negatively impacted by atherosclerosis. Secondly, ageing is a major risk factor for higher incidences of chronic disorders such as cardiovascular diseases (CVDs), neurodegenerative diseases, metabolic diseases, musculoskeletal diseases and immune-senescence diseases [24, 25]. Furthermore, spermidine protects against cardiac aging by enhancing left ventricular elasticity, diastolic function, and mitochondrial function [26]. Additionally, It had been demonstrated that CAD, essential hypertension, and heart failure are highly impacted by spermidine levels [27]. Remarkably, a recent animal study stated that spermidine’s ameliorating effect on spermatogenic disorder in the testis of mice with type 1 diabetes mellitus occurred through enhancing spermatogenic cell proliferation and activating the glycolytic pathway [28].

Another major finding of the current study was the presence of varicocele in all patients. About 84% of the included patients had clinical grade 2 and 3 varicocele. It was well established that varicocele could negatively affect endoplasmic reticulum and the unfolded protein response, which accelerated cell apoptosis [29]. Furthermore, varicocele was associated with increased sperm antibodies [30], abnormal testosterone secretion and defective spermatogenesis [31] and increased cytokines mediated inflammation [32]. Numerous studies had demonstrated that the SRR were notably greater in cases of hypospermatogenesis compared to mixed pathologies such as SCO syndrome and spermatogenic foci, as well as primary spermatocyte arrest [33–35]. These findings are consistent with our own findings. Interestingly, the current study shed light on a neglected sector of infertile patients who suffered from secondary azoospermia as a cause of male infertility with the utmost importance to explore the risk factors that led to such condition through implementing future studies that deploy larger cohorts of such group of patients.

### Limits of the study

Our investigation was limited by its small sample size that could be regarded as the major limitation of the current work. However, this was inevitable given the rarity of our study’s targeted population. Furthermore, inability to evaluate obesity of the included cases should be seen as another limitation of the current study. Moreover, inability to do paternity test was seen as a further limitation. However, it’s applicability in the current study would be questionable as it would lead to major social and interralationships instability as well as it would be rejected by the included patients. Finally, inability to evaluate the impact of the advanced paternal age represented by the current study on the health of the offspring.

However, it should be noted that there are no current precautions that can minimize the risks of advanced paternal age, nor available specific tests that can recognize individuals at increased risk [22].

## Conclusion

It appears that the etiopathogenesis of secondary azoospermia are multifactorial. Varicocele and CAD are major factors to be considered. Future studies should be implemented deploying larger pools of patients suffering from the same condition to affirm the findings of this primary study.

## Data Availability

The data that support the findings of this study are available from the corresponding author upon reasonable request.

## Abbreviations

CAD: Coronary artery disease
CLIA: Chemiluminescence immunoassay
FSH: Follicle stimulating hormone
LH: Leutinizing hormone
MA: Maturation arrest
microTESE: Microsurgical testicular sperm extraction
NOA: Non obstructive azoospermia
SCO syndrome: Sertoli cell only syndrome
SRR: Sperm retrieval rate

## References

[1] Benksim A, Elkhoudri N, Ait Addi R, Baali A, Cherkaoui M. Difference between primary and secondary infertility in Morocco: frequencies and associated factors. Int J Fertil Steril. 2018;12(2):142–6. 10.22074/IJFS.2018.5188. 29707931 10.22074/IJFS.2018.5188 PMC5936612

[2] Katib AA, Al-Hawsawi K, Motair W, Bawa AM. Secondary infertility and the aging male, overview. Cent Eur J Urol. 2014;67(2):184. 10.5173/CEJU.2014.02.ART13. 10.5173/CEJU.2014.02.ART13 PMC4132591 25140235

[3] Gorelick JI, Goldstein M. Loss of fertility in men with varicocele. Fertil Steril. 1993;59(3):613–6. 10.1016/S0015-0282(16)55809-9. 8458466 10.1016/S0015-0282(16)55809-9

[4] Johnson SL, Dunleavy J, Gemmell NJ, Nakagawa S. Consistent age-dependent declines in human semen quality: a systematic review and meta-analysis. Ageing Res Rev. 2015;19:22–33. 10.1016/j.arr.2014.10.007. Elsevier Ireland Ltd. 25462195 10.1016/j.arr.2014.10.007

[5] Dias TR, Alves MG, Bernardino RL, Martins AD, Moreira AC, Silva J, Barros A, Sousa M, Silva BM, Oliveira PF. Dose-dependent effects of caffeine in human Sertoli cells metabolism and oxidative profile: relevance for male fertility. Toxicology. 2015;328:12–20. 10.1016/J.TOX.2014.12.003. 25486098 10.1016/J.TOX.2014.12.003

[6] Hayden RP, Flannigan R, Schlegel PN. The role of lifestyle in male infertility: diet, physical activity, and body habitus. Curr Urol Rep. 2018;19(7):56. 10.1007/S11934-018-0805-0. 29774489 10.1007/S11934-018-0805-0

[7] Hamada AJ, Esteves SC, Agarwal A. A comprehensive review of genetics and genetic testing in azoospermia. Clinics. 2013;68(Suppl 1):39. 10.6061/CLINICS/2013(SUP01)06. 23503954 10.6061/CLINICS/2013(SUP01)06 PMC3583155

[8] UW Health. Infertility: generations fertility care. University of Wisconsin Hospitals and Clinics Authority. https://www.uwhealth.org/infertility. Accessed 6 Mar 2021.

[9] World Medical Association. World Medical Association Declaration of Helsinki: ethical principles for medical research involving human subjects. JAMA. 2013;310(20):2191–4. 10.1001/jama.2013.281053. 24141714 10.1001/jama.2013.281053

[10] World Health Organization (WHO). WHO laboratory manual for the examination and processing of human semen. 5th ed. Geneva: WHO; 2010. p. 271.

[11] Agarwal A. Abstinence time and its impact on basic and advanced semen parameters. Urology. 2016;94:102–10. 10.1016/j.urology.2016.03.059. 27196032 10.1016/j.urology.2016.03.059

[12] Cerilli LA, Kuang W, Rogers D. A practical approach to testicular biopsy interpretation for male infertility. ArccPathol Lab Med. 2010;134:1197–204. 10.5858/2009-0379-RA.1. 10.5858/2009-0379-RA.1 20670143

[13] Li H, Chen LP, Yang J, Li MC, Chen RB, Lan RZ, Wang SG, Liu JH, Wang T. Predictive value of FSH, testicular volume, and histopathological findings for the sperm retrieval rate of microdissection TESE in nonobstructive azoospermia: a meta-analysis. Asian J Androl. 2018;20(1):30–6. 10.4103/aja.aja_5_17. (PMID: 28361811; PMCID: PMC5753551). 28361811 10.4103/aja.aja_5_17 PMC5753551

[14] Gill K, Jakubik-Uljasz J, Rosiak-Gill A, Grabowska M, Matuszewski M, Piasecka M. Male aging as a causative factor of detrimental changes in human conventional semen parameters and sperm DNA integrity. Aging Male. 2021;23(5):1321–32. 10.1080/13685538.2020.1765330. 10.1080/13685538.2020.1765330 32425138

[15] Verón GL, Tissera AD, Bello R, Beltramone F, Estofan G, Molina RI, Vazquez-Levin MH. Impact of age, clinical conditions, and lifestyle on routine semen parameters and sperm kinematics. Fertil Steril. 2018;110(1):68–e754. 10.1016/j.fertnstert.2018.03.016. 29980266 10.1016/j.fertnstert.2018.03.016

[16] Frattarelli JL, Miller KA, Miller BT, Elkind-Hirsch K, Scott RT. Male age negatively impacts embryo development and reproductive outcome in donor oocyte assisted reproductive technology cycles. Fertil Steril. 2008;90(1):97–103. 10.1016/J.FERTNSTERT.2007.06.009. 17765235 10.1016/J.FERTNSTERT.2007.06.009

[17] Humm KC, Sakkas D. Role of increased male age in IVF and egg donation: is sperm DNA fragmentation responsible? Fertil Steril. 2013;99(1):30–6. 10.1016/J.FERTNSTERT.2012.11.024. 23273987 10.1016/J.FERTNSTERT.2012.11.024

[18] Pino V, Sanz A, Valdés N, Crosby J, Mackenna A. The effects of aging on semen parameters and sperm DNA fragmentation. JBRA Assist Reprod. 2020;24(1):82. 10.5935/1518-0557.20190058. 31692316 10.5935/1518-0557.20190058 PMC6993171

[19] Stone BA, Alex A, Werlin LB, Marrs RP. Age thresholds for changes in semen parameters in men. Fertil Steril. 2013;100(4):952–8. 10.1016/j.fertnstert.2013.05.046. 23809502 10.1016/j.fertnstert.2013.05.046

[20] Ramasamy R, Trivedi NN, Reifsnyder JE, Palermo GD, Rosenwaks Z, Schlegel PN. Age does not adversely affect sperm retrieval in men undergoing microdissection testicular sperm extraction. Fertil Steril. 2014;101(3):653–5. 10.1016/j.fertnstert.2013.11.123. (Epub 2014 Jan 11 PMID: 24424360). 24424360 10.1016/j.fertnstert.2013.11.123

[21] Amer MK, Ahmed AR, Abdel Hamid AA, GamalEl Din SF. Can spermatozoa be retrieved in non- obstructive azoospermic patients with high FSH level? A retrospective cohort study. Andrologia. 2019;51:e13176. 10.1111/and.13176. 30311269 10.1111/and.13176

[22] Martins da Silva S, Anderson RA. Reproductive axis ageing and fertility in men. Rev Endocr Metab Disord. 2022;23(6):1109–21. 10.1007/s11154-022-09759-0. (Epub 2022 Nov 2. PMID: 36322295; PMCID: PMC9789007). 36322295 10.1007/s11154-022-09759-0 PMC9789007

[23] Haverich A. A surgeon’s view on the pathogenesis of atherosclerosis. Circulation. 2017;135(3):205–7. 10.1161/CIRCULATIONAHA.116.025407. (PMID: 28093492). 28093492 10.1161/CIRCULATIONAHA.116.025407

[24] Ni YQ, Zhan JK, Liu YS. Roles and mechanisms of MFG-E8 in vascular aging-related diseases. Ageing Res Rev. 2020;64:101176. 10.1016/j.arr.2020.101176. (Epub 2020 Sep 21. PMID: 32971257). 32971257 10.1016/j.arr.2020.101176

[25] Ni YQ, Lin X, Zhan JK, Liu YS. Roles and functions of exosomal non-coding RNAs in vascular aging. Aging Dis. 2020;11:164–78. 10.14336/AD.2019.0402. (PMID: 32010490; PMCID: PMC6961769). 32010490 10.14336/AD.2019.0402 PMC6961769

[26] Eisenberg T, Abdellatif M, Schroeder S, Primessnig U, Stekovic S, Pendl T, et al. Cardioprotection and lifespan extension by the natural polyamine spermidine. Nat Med. 2016;22:1428–38. 10.1038/nm.4222. (Epub 2016 Nov 14. PMID: 27841876; PMCID: PMC5806691). 27841876 10.1038/nm.4222 PMC5806691

[27] Soda K, Kano Y, Chiba F. Food polyamine and cardiovascular disease–an epidemiological study. Glob J Health Sci. 2012;4:170–8. 10.5539/gjhs.v4n6p170. (PMID: 23121753; PMCID: PMC4776963). 23121753 10.5539/gjhs.v4n6p170 PMC4776963

[28] Wang JY, Ma D, Luo M, Tan YP, Ou Zhong, Tian G, Lv YT, Li MX, Chen X, Tang ZH, Hu LL, Lei XC. Effect of spermidine on ameliorating spermatogenic disorders in diabetic mice via regulating glycolysis pathway. Reprod Biol Endocrinol. 2022;20(1):45. 10.1186/s12958-022-00890-w. (PMID: 35255928; PMCID: PMC8900360). 35255928 10.1186/s12958-022-00890-w PMC8900360

[29] Hosseini M, Shaygannia E, Rahmani M, Eskandari A, Golsefid AA, Tavalaee M, et al. Endoplasmic Reticulum stress (ER stress) and Unfolded Protein Response (UPR) occur in a rat varicocele testis model. Oxid Med Cell Longev. 2020;2020:5909306. 10.1155/2020/5909306. 32802266 10.1155/2020/5909306 PMC7411497

[30] Tchiokadze S, Galdava G. Humoral immunity status if infertile men antisperm antibodies and various pathologies of reproductive organs. Georgian Med News. 2015;241:58–62 (PMID: 25953941). 25953941

[31] Oh YS, Jo NH, Park JK, Gye MC. Changes in inflammatory cytokines accompany deregulation of claudin-11, resulting in inter-sertoli tight junctions in varicocele rat testes. J Urol. 2016;196(4):1303–12. 10.1016/j.juro.2016.05.004. 27164517 10.1016/j.juro.2016.05.004

[32] Finelli R, Pallotti F, Cargnelutti F, Faja F, Carlini T, Rizzo F, et al. Sperm DNA damage and cytokines in varicocele: a case-control study. Andrologia. 2021;53(5):e14023. 10.1111/and.14023. 33689198 10.1111/and.14023

[33] Cito G, Coccia ME, Dabizzi S, Morselli S, Della Camera PA, Cocci A, et al. Relevance of testicular histopathology on prediction of sperm retrieval rates in case of non-obstructive and obstructive azoospermia. Urologia. 2018;85(2):60–7 (Epub 2018 Mar 23. PMID: 29846141). 29846141 10.1177/0391560318758940

[34] Guler I, Erdem M, Erdem A, Demirdağ E, Tunc L, Bozkurt N, Mutlu MF, Oktem M. Impact of testicular histopathology as a predictor of sperm retrieval and pregnancy outcome in patients with nonobstructive azoospermia: correlation with clinical and hormonal factors. Andrologia. 2016;48(7):765–73. 10.1111/AND.12510. 26688565 10.1111/AND.12510

[35] Sousa M, Fernandes S, Barros A. Prognostic factors for successful testicle spermatid recovery. Mol Cell Endocrinol. 2000;166(1):37–43. 10.1016/S0303-7207(00)00295-1. 10989206 10.1016/S0303-7207(00)00295-1

